# Life Cycle Assessment-Based Carbon Footprint Accounting Model and Analysis for Integrated Energy Stations in China

**DOI:** 10.3390/ijerph192416451

**Published:** 2022-12-08

**Authors:** Xiaorong Sun, Xueping Pan, Chenhao Jin, Yihan Li, Qijie Xu, Danxu Zhang, Hongyang Li

**Affiliations:** 1 College of Energy and Electrical Engineering, Hohai University, Nanjing 211100, China; 2 Business School, Hohai University, Nanjing 211100, China; 3 Research & Development, GE Digital, Bothell, WA 98011, USA

**Keywords:** carbon footprint, integrated energy station, lifecycle assessment, renewable energy

## Abstract

To achieve its carbon neutrality goal, China has invested broadly in energy infrastructure and the emerging integrated energy stations (IESs) projects will bring enormous opportunities. Accurate carbon emission accounting for IESs is challenging in view of the complexity of the manufacturing process and uncertainty in construction and operation processes. To overcome these challenges, this paper develops a novel quantitative carbon footprint analysis model for IESs from a lifecycle perspective, with production and materialization, construction, operation and maintenance, and disposal and recycling phases considered. The method is applied on a 110 kV wind power IES project in China, to analyze and calculate lifecycle carbon emissions, identify the key influence factors of carbon footprints and provide suggestions for carbon reduction. The findings can identify key influence factors and provide suggestions for carbon reduction for the development of IES projects.

## 1. Introduction

Climate change is an urgent global challenge with a long-term impact on the sustainable development of human society. China, as an important participant and practitioner in addressing global climate change, has claimed it will meet its carbon peaking goal in 2030 and its carbon neutrality goal in 2060. The development of low-carbon energy industry is important and necessary in view of its critical role in low-carbon energy transition.

The integrated energy station (IES) synthesizes the design, construction, management, and operation of multiple stations including energy supply stations, energy service stations, energy storage stations, electric vehicle charging stations, data centers, 5G base stations, etc. It has become a complicated multi-station integrated energy system providing comprehensive energy services to meet the needs of different end-users. As one of the practical application scenarios of the regional integrated energy system, the IES has become an important development direction within integrated energy services, and has been proved to be an efficient approach to adapt to China’s national conditions [1,2,3,4].

Currently, there are two types of IES projects in China: new construction of wind-solar-storage-charging IESs [5,6], and the renovation of conventional substations. By integrating independent wind farms or photovoltaic power stations with energy storage and charging stations, the wind-solar-storage-charging IES is suitable for large-scale centralized locations, such as fast charging stations, business parks, and commercial residences. The Pacific Northwest National Laboratory is working on the rural area distributed wind integration network development project to understand, address, and reduce the technical risks and market barriers to distributed wind adoption by rural utilities with a similar concept to wind power IES in China [7]. European countries are also involved in the development of distributed energy including wind power in the process of transition to clean energy [8]. The energy storage system in wind-solar-storage-charging IES charges electricity during low-peak periods and supports fast charging load during peak periods, bringing the benefits of local consumption of the distributed energy generation, reduction in the load difference, and improvement in system operation efficiency. The other practical application of IESs is the transformation and renovation of existing conventional substations. Considering different applicable scenarios, two pathways of physical stations and virtual stations were put forward from the perspective of construction planning and site transformation to realize the upgrade of traditional substations to IESs [9].

IESs, as physical hubs to improve renewable energy consumption, reduce carbon emissions and improve energy utilization efficiency, will play an important role in the future energy system and its carbon emission accounting should be fully considered. However, most of the current research on carbon emissions of IESs focus on the optimal operation or energy storage capacity configuration. As a critical and prerequisite step before planning and operation, carbon emission calculation and analysis of IESs require appropriate accounting models.

Lifecycle assessment (LCA) theory has been widely used in the research on carbon emissions for a single product or project such as a conventional substation, public building, wind turbine, and rooftop photovoltaic system [10,11,12,13]. Since the IES is a holistic system, it is urgent to develop a carbon emission accounting model in a systematic way from an overview perspective, analyzing the main carbon emissions affecting factors, and provide theoretical guidance and countermeasures for emission reduction actions.

To address the above issues, this paper constructs a novel carbon footprint calculation and analysis model based on LCA for multi-station IESs, with production and materialization, construction, operation and maintenance, and disposal and recycling phases considered. The method is applied on a 110 kV wind power IES project in China to calculate and analyze lifecycle carbon emissions, identify key affecting factors, and provide suggestions for carbon reduction. Through a combination of quantitative modeling and qualitative analysis, this paper aims to provide guidance and suggestions for the planning, construction, and operation of IESs for energy and electricity industries.

## 2. Literature Review on Carbon Footprint Analysis

### 2.1. Carbon Footprint Analysis Methods

Carbon footprint is the carbon equivalent conversion between carbon dioxide and other greenhouse gases based on the global warming trend under the influence of sustainable development [14]. Carbon footprint analysis methods mainly include the input–output method, emission factor method, and LCA method. Each method has its own advantages and disadvantages [15].

#### 2.1.1. Input-Output Method

Input–output analysis is a top-down calculation method, which reflects the quantitative dependence of input and output among various departments in the economic system [16]. The core idea of input–output analysis is to characterize the physical conversion relationship between output and input through the consumption coefficient matrix. It avoids system boundary setting, comprehensively reflects the direct and indirect carbon emission relationship of various departments, and overcomes repeated or missing calculations caused by the complex production relationship among departments. However, the method requires data obtained from broad scales, and takes a lot of time and effort to derive input–output tables. Moreover, the input–output method can only be applied to macro-level carbon footprint analysis, such as government, industry sectors, and enterprises, and there are major obstacles at the micro level [17].

#### 2.1.2. Emission-Factor Method

Emission-factor approach is a carbon emission accounting method proposed by the United Nations Climate Change Committee, which describes the specific calculation of greenhouse gases [18]. The main idea is to construct the activity level (the amount of carbon emission source consumption) and the emission factor (the amount of greenhouse gases produced by a unit of carbon emission source) for each emission source according to the list of carbon emission inventories, and then calculate the product of the activity level and the emission factor. The common formula used in the emission-factor approach is:

Carbon emissions = Activity level × Emission factor (1)

The advantage of the method is that it could comprehensively examine the greenhouse gas emissions caused by the combustion of different fossil fuels, and the calculation process is relatively simple. However, the method could not cover the implied indirect carbon emissions and usually requires rectification when applied at the meso and micro levels. In addition, due to differences in production technology levels and energy quality in various regions, it is difficult to select regional emission factors.

#### 2.1.3. LCA Method

LCA is a bottom-up process-based analysis method, which considers the greenhouse gas emissions from cradle to grave in the whole lifecycle process of the product or service obtained from raw materials, production, usage and disposal [19,20]. With the concept of carbon footprint, LCA has become the most important carbon footprint accounting method at the micro level, especially at product or process level.

Carbon footprint accounting based on the LCA method considers both the direct and indirect carbon emissions of the system during the lifecycle, and it thus typically provides high accuracy. The method is suitable for accounting for carbon footprint at the micro level such as products, but there are major obstacles in the acquisition of macro level data. Furthermore, uncertainty is the main problem faced by this method, such as the truncation error in the process of system boundary delineation and the lack of localized characteristic factors in LCA phases.

### 2.2. Carbon Footprint Analysis for IESs

Research on the carbon footprint of IESs is particularly important for evaluating the benefits of energy and emission reduction. Carbon footprint analysis is the basis for the planning and construction of IESs and low-carbon operation. Setting both objective calculation and carbon emission constraint in the optimization model requires calculating the carbon footprints of the system [21,22,23,24]. The relevant literature is summarized in Table 1 and reviewed below.

Much research has been conducted on carbon emission calculation and analysis from the power generation system level via considering carbon objective or carbon constraints in the optimization models. A carbon emission calculation for energy supply entities with different carbon emission levels based on the emission factor method is developed in [25]. The authors in [26] further considered the influence of the energy supply low-carbon transformation process on the carbon emission factor. Recently, carbon flow-based methods have been widely used when accounting for carbon emissions within a network of multiples nodes. References [27,28] proposed a carbon flow analysis method based on carbon emission factor and power energy flow to consider system operating characteristics and network characteristics.

IES is generally one entity with multiple components or modules in the distribution system; carbon footprint analysis based on LCA is suitable to reflect carbon emissions at the product level. LCA-based carbon footprint analysis research has been carried out for partial components or specific parts of the IESs, such as substations, transformer equipment, batteries or buildings.

Reference [29] employed the LCA method to quantify carbon emissions with the typical design scheme of the 110 kV substation. It provided plans to reduce substation carbon emissions of carbon reduction equipment, energy supply substitution, energy-saving building, and optimization operation. Carbon emissions of various substations and transformers were analyzed and compared in [30] by the LCA method with inventory data from a global manufacturer. The study suggested carbon emissions could be significantly reduced by reducing power losses and SF_6_ leakages in gas insulated switchgear (GIS). The LCA-based studies on solar photovoltaic technologies, such as silicon, thin film, dye-sensitized solar cell, perovskite solar cell, and quantum dot-sensitized solar cell, were reviewed and analyzed in [31]. The employed LCA methods started from the extraction of raw materials until the disposal or recycling of the solar photovoltaic. In [32], the LCA method was utilized to evaluate the environmental impact and energy benefit of offshore wind power. The study assumed that the lifecycle of offshore wind power had four stages: production, installation, operation and maintenance, and end-of-life, and found the largest environmental impact was attributable to the use of ferrous metal, and waste recycling could significantly reduce carbon emissions. A cradle-to-cradle LCA method was developed in [33] to investigate the carbon footprint of a lithium-ion battery in China under the reality and future scenarios. Carbon footprint in the full lifespan including battery production considering raw materials, use, recycling, and remanufacturing stages, were investigated in detail. In [34], three streams of LCA methods including lifecycle assessment, lifecycle energy assessment, and lifecycle carbon emissions assessment were applied and compared for buildings. The input, output and functional units of each method were described and analyzed to support the improvement of decision making in green buildings.

**Table 1:** Literature of carbon emission analysis for IESs.

Method	Level	Subject	Reference
Emission factor	System level	Carbon objective or carbon constraints	[25,26]
	Network level	Distribution network	[27,28]
LCA	Product level	Substation or transformer	[29,30]
		Solar photovoltaic station	[31]
		Wind power station	[32]
		Lithium-ion battery	[33]
		Building	[34]

The above study brings insight to the carbon footprint analysis for IES components. However, there are few studies about carbon footprint accounting standards and accounting models for IESs as an entity. Based on existing studies, this paper takes the IES as a holistic system, and analyzes the main factors affecting carbon emissions from the perspective of the whole process of production, construction, operation and recycling. This paper aims to construct a quantitative carbon footprint calculation and analysis method for IESs. The LCA method is used to analyze the carbon footprint of power transformation, energy storage, distributed generation, charging and other modules in the IESs. Thus, with energy consumption level obtained and corresponding carbon emission factors collected, the carbon emissions of each phase, each module, and each equipment in the IES can be accurately calculated.

## 3. Carbon Footprint Accounting for IESs Based on LCA

According to the definition of ISO 14044 standard, using the LCA method to calculate the carbon footprint of a service or product includes four steps: goal and scope definition, inventory analysis, impact assessment, and result interpretation.

### 3.1. Goal and Scope

**Figure 1:**
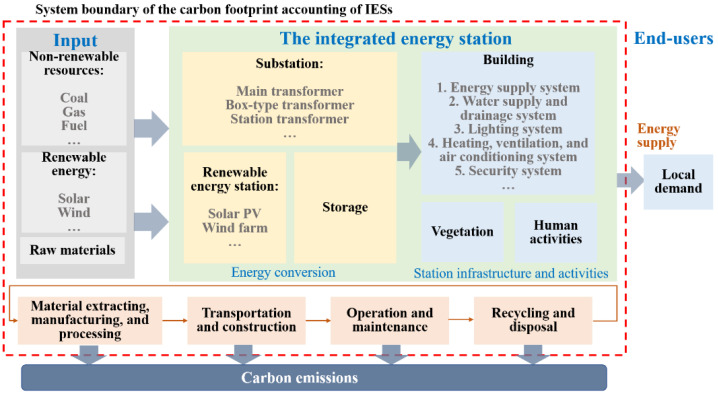
System boundary of the IES carbon emission accounting.

The research scope is to quantify the carbon footprint of the IESs, calculate and discuss the carbon emission distribution at different phases of the lifecycle, and provide guidance or suggestions for the planning, construction and operation of IESs to reduce carbon emission. The system boundary of the carbon footprint accounting for the IESs is shown in Figure 1. A functional unit characterizes a quantity of a product or product system based on the functionality. In this paper, the substation, renewable energy station, storage and building in the IESs are taken as the study objects. The system takes input from fossil and renewable energy sources to generate electricity and fossil fuel combustion, into the equipment or building, and outputs energy and carbon emissions. The LCA-based carbon footprint accounting for IESs includes the acquisition of raw materials, production, transportation, energy conversion, and energy terminal consumption, and recycling and disposal within the life span.

In this paper, land resources and human activities are considered as essential factors within the life span of IESs. Vegetation destruction, vegetation restoration, and energy/fuel consumption caused by human activities are thus considered within the system boundary.

### 3.2. Inventory Analysis

**Figure 2:**
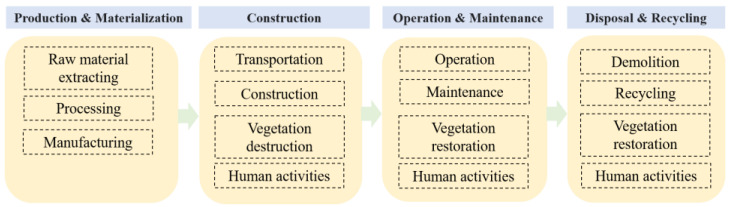
LCA phases of carbon footprint analysis for integrated energy stations.

Inventory analysis is a detailed quantitative analysis of the energy consumption and carbon emissions for each module and equipment throughout the lifecycle. Figure 2 shows the phase division of the lifecycle for IESs. 

Land resources impact is considered in the construction, operation and maintenance, and disposal and recycling phases. The construction phase results in carbon sink due to vegetation damage temporarily and permanently occupied by the IES project. The operation and maintenance phase brings carbon sink loss due to vegetation destruction permanently occupied by the IES project and carbon sink from greens planted in the station. After the demolition in the disposal and recycling phase, occupied land is recovered to the original type.

Human activities are also considered in the construction, operation and maintenance, and disposal and recycling phases. Human activities in construction and disposal and recycling phases include the on-site activities and commuting of construction workers. Human activities in operation and maintenance phase may include the working, living and commuting of the on-duty staff in the IES station.

#### 3.2.1. Production and Materialization Phase

Carbon emissions in the production and materialization phase include carbon emissions generated during the process of the equipment or products required for the construction of an IES, from the production of raw materials to the manufacture and assembly of accessories, into a complete product or equipment. The total amount of carbon emissions in the production and materialization stage *CE_pe_* is the sum of all production raw material consumption and materialization energy consumption carbon emissions:

(2) $$\begin{array}{l} CE_{pe}=CE_{p}+CE_{e}\\ \;\left\{ \begin{array}{l} CE_{p}=\sum_{n=1}^{N1}Q_{p,n}EF_{p,n}\\ CE_{e}=\sum_{n=1}^{N2}Q_{e,n}EF_{e,n} \end{array}\right. \end{array}$$

where *CE_p_* is the carbon emission in the production stage, and *CE_e_* is the carbon emission in the materialization stage. *N*1 is the total number of types of materials used in the production equipment. *Q_p,n_* and *EF_p,n_* is the amount level and carbon emission factor of the *n*th material used in the production equipment, respectively. *N*2 is the total number of energy types used by the materialization equipment. *Q_e,n_* and *EF_e,n_* is the energy level and carbon emission factor of the *n*th energy used in materialization, respectively.

#### 3.2.2. Construction Phase

The construction phase is the process of various resources (including materials, machinery, energy, technology, etc.) put into the project construction in accordance with relevant standards and rationally organized in time and space to become an IES entity. Carbon emissions at this stage include carbon emissions from equipment transportation and mechanical installation of products or equipment.

The total carbon emission *CE_c_* at this phase is the sum of the carbon emission *CE_t_* in the transportation process, the carbon emission of the construction energy consumption *CE_ce_*, the carbon emission of the earthworks *CE_ew_*, the carbon emission Δ*CE_v_* indirectly caused by the change in the vegetation carbon sink, and the carbon emission *CE_ch_* of human activities during construction:

(3) $$\begin{array}{l} CE_{c}=CE_{t}+CE_{ce}+CE_{ew}+△CE_{v}+CE_{ch}\\ \;\left\{ \begin{array}{l} CE_{t}=\sum_{n=1}^{N3}Q_{t,n}L_{t,n}EF_{t,n}\\ CE_{ce}=\sum_{n=1}^{N4}Q_{ce,n}EF_{ce,n}\\ CE_{ew}=V\cdot f_{ew}EF_{ew}\\ △CE_{v}=\sum_{n=1}^{N5}S_{v,n}f_{v,n}T_{c} \end{array}\right. \end{array}$$

where *N*3 is the total number of transportation types of the transported parts and materials; *Q_t,n_* is the total weight of parts carried in the *n*th transportation type; *L_t,n_* is the transportation distance; and *EF_t,n_* is the carbon emission factor of the *n*th transportation type. *N*4 is the total number of energy types used in the construction. *Q_ce,n_* and *EF_ce,n_* is the energy consumption level and the carbon emission factor of the *n*th energy consumed in the construction stage, respectively. *V* is the earthwork quantity; *f_ew_* is the earthwork energy consumption coefficient; and *EF_ew_* is the carbon emission factor of the earthwork energy consumption. *N*5 is the total number of damaged vegetation types; *S_v,n_* is the original area of the *n*th vegetation in the construction land; *f_v,n_* is the unit carbon sequestration of the *n*th vegetation; and *T_c_* is the construction period.

#### 3.2.3. Operation and Maintenance Phase

Carbon emissions in the operation and maintenance phase include: (1) carbon emissions from energy consumption during the operation of IES’s products or equipment; (2) carbon emissions from energy consumption to maintain the normal operation of equipment; (3) carbon emissions from heating, ventilation, air conditioning, lighting and other equipment; (4) carbon emissions indirectly caused by changes in vegetation carbon sinks caused by vegetation destruction; (5) the carbon emissions reduction caused by green vegetation; and (6) the carbon emission of human activities during operation and maintenance. Then the carbon emissions *CE_om_* of the IESs during the operation and maintenance phase is obtained by accumulating carbon emissions caused by different energy consumption levels of each module:

(4) $$\begin{array}{l} CE_{om}=CE_{o}+CE_{m}+△CE_{ov}-△CE_{op}+CE_{oh}\\ \;\left\{ \begin{array}{c} CE_{o}=E\cdot EF\\ CE_{m}=CE_{mp}+CE_{mt}\\ \;CE_{mp}=\sum_{n}^{N6}Q_{mp,n}EF_{mp,n}\\ \;CE_{mt}=\sum_{n}^{N7}Q_{mt,n}L_{mt,n}EF_{mt,n}\\ △CE_{ov}=\sum_{n}^{N8}S_{ov,n}f_{ov,n}T_{o}\\ △CE_{op}=\sum_{n}^{N9}S_{op,n}f_{ov,n}T_{o} \end{array}\right. \end{array}$$

where *CE_o_* is the carbon emission generated by the operation of the IES; *CE_m_* is the carbon emission generated by the maintenance work; Δ*CE_ov_* is the carbon emission indirectly caused by the change in the carbon sink caused by the destruction of the vegetation during the operation phase; Δ*CE_op_* is the carbon sink from planting green vegetation during operation and maintenance. *E* is the power consumption level of equipment operation; and *EF* is the carbon emission factor of the local electrical grid. *CE_mp_* is the carbon emission in the production phase of the parts required for the maintenance phase; *CE_mt_* is the emission in the transportation phase of the parts required in the maintenance phase. *N*6 is the total number of types of parts used in the maintenance phase; *Q_mp_*_,*n*_ and *EF_mp,n_* is the weight and the carbon emission factor of the *n*th type of parts used in the maintenance phase. *N*7 is the total number of transportation types; *Q_mt_*_,*n*_ is the component weight of the *n*th transportation mode; *L_mt,n_* is the transportation distance; and *EF_mt,n_* is the carbon emission factor of the *n*th transportation mode. *N*8 is the number of damaged vegetation types; *S_ov,n_* is the original area of the *n*th vegetation on the project land; *f_ov,n_* is the unit carbon sequestration of the *n*th vegetation; and *T_o_* is the operation and maintenance time. *N*9 is the number of green vegetation species; and *S_op,n_* is the area of the *n*th vegetation.

#### 3.2.4. Disposal and Recycling Phase

Carbon emission *CE_r_* in the disposal and recovery phase includes: (1) carbon emission *CE_re_* generated due to the use of mechanical equipment and other equipment during demolition of IES facilities; (2) carbon emission reduction *CE_rp_* generated by the recycling, incineration, landfill and other disposal of waste materials; (3) carbon emission Δ*CE_dp_* of vegetation recoveries to original land; and (4) carbon emission *CE_dh_* of human activities during dismantling.

(5) $$\begin{array}{l} CE_{r}=CE_{re}-CE_{rp}-\;△CE_{dp}+\;CE_{dh}\\ \;\left\{ \begin{array}{l} CE_{re}=\sum_{n}^{N10}Q_{re,n}EF_{re,n}\\ \;CE_{rp}=\sum_{n}^{N11}Q_{rp,n}EF_{rp,n} \end{array}\right. \end{array}$$

where *N*10 is the total number of types of energy used for dismantling and disposal; *Q_re,n_* and *EF_re,n_* is the consumption level and carbon emission factor of the *n*th energy used in dismantling and disposal, prospectively. *N*11 is the number of types of recycled materials; *Q_rp,n_* and *EF_rp,n_* is the weight and the carbon emission factor of the *n*th material recycled.

#### 3.2.5. Lifecycle Carbon Emissions

The Lifecycle Carbon Emission (*LCCE*) of an IES is the sum of the carbon emissions in the four phases of production and materialization, construction, operation and maintenance, and disposal and recovery, which is expressed as:

(6) $$LCCE=CE_{pe}+CE_{c}+CE_{om}+CE_{r}$$

## 4. Case Analysis

### 4.1. Project Overview

The study case project is a wind power IES, located in Henan Province, China. The construction of the project started in July 2021, and all electrical facilities were connected to the grid in December 2021. The life span of this wind power IES is 20 years for the case study.

**Figure 3:**
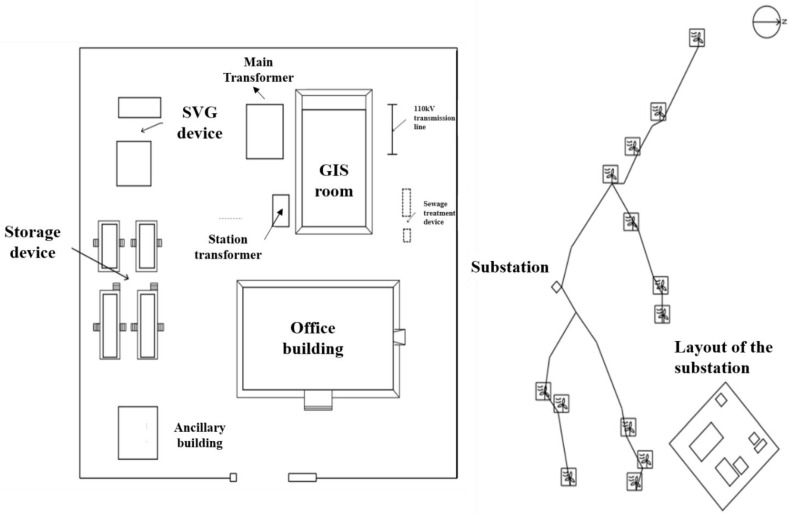
Structure of the wind power IES.

This IES project consists of a 110 kV substation and a wind farm. Wind turbines were installed on a mountainous terrain, utilizing wind energy resources and providing clean and renewable energy to the power grid. The total installation capacity is 42 MW, which is capable of providing a maximum of 107,069 MWh of clean energy to the grid each year, with an annual equivalent of 2549 h of full load. The substation consists of a 50 MVA main transformer, grounding transformers and station transformers, a comprehensive office building, ancillary buildings, prefabricated cabins for primary and secondary electrical equipment, 4.4 MWh energy storage devices, static var generator (SVG) device, GIS room, etc. The structure of the wind power IES is shown in Figure 3.

The carbon footprint analysis of the wind power IES considers the following modules:

- The transformer module includes a 50 MVA main transformer with a voltage level of 110/35 kV, a 35 kV integrated circuit, 14 box-type transformers, and a station transformer.
- The wind farm module includes 14 wind turbines with single turbine capacity 3 MW (Envision EN156-3.0, 3 MW), which are connected to the low-voltage side feeder cabinet of the box-type transformer, and then connected to the 35 kV distribution network. The wind turbine adopts a steel cone structure with a hub height of 100 m.
- The energy storage module uses a prefabricated cabin-type lithium iron phosphate battery with a capacity of 4.4 MWh. The scale of the energy storage module is based on the output of the wind farm module and combined with the demand for peak regulation and frequency regulation of the power grid.
- The building module includes the control building, 35 kV power distribution room and auxiliary room. The specific parameters of the building are shown in Table 2. The building structure is reinforced concrete with clay porous brick.
- The vegetation module includes the loss of carbon sinks caused by vegetation damage during construction and operation, and the compensation of carbon sinks for green plants in the station.
- The human activity module includes the energy consumption for on-site living and commuting during the construction process and the energy consumption and commuting of the on-duty personnel during operation and maintenance.

### 4.2. Data Collection and Computational Analysis

The following step collects data and analyzes carbon emissions in each phase for each module. Based on the design and parameters in the actual construction and operation of the wind power IES, corresponding project content and carbon emission factors are collected, and carbon emissions of the substation, wind farm, building, energy storage and other modules are thus calculated.

Table 3 summarizes the carbon footprint sources at each phase of the lifecycle for each module. Table 4 shows the carbon emission factors collected for the main materials and energy used in each module. Due to the limited material data available, some of the data used in this paper have been converted reasonably. For example, the materials used in the transformer in the substation module and power consumption are converted according to the capacity of the transformer, and the materials usage in the building module and the energy consumption are calculated based on the building area and height.

#### 4.2.1. Calculation and Analysis in the Production and Materialization Phase

Carbon footprints in this phase include carbon emissions of the product or equipment from raw materials to the manufacture and assembly of accessories. Carbon emission sources in the transformer module are composed of raw materials to produce transformer core, coil, fuel tank, secondary equipment such as protection devices, voltage regulators, and the energy consumed in the manufacturing process. Carbon emissions of the wind farm module result from wind turbine part production and assembly (such as tower bases, towers, nacelles, generators, hubs, blades etc.), as well as the energy consumption required for production and assembly. Carbon emission resources in the building module consist of the production of materials such as cement, steel, sand, and brick. Carbon emission of the energy storage module is generated by lithium iron phosphate battery materials, the energy consumption during the assembly and molding process, as well as the production of prefabricated cabin. According to [39], the carbon emissions of producing 1 MWh lithium iron phosphate battery is 216 t CO_2_e, thus the manufacture and production of a 4.4 MWh storage battery is 950.4 t. The storage system is in a prefabricated cabin with an estimated steel weight of 37.45 t.

With the above collected data, detailed carbon emissions in the production and materialization phase are obtained in Table 5. As can be seen, carbon emissions of the transformer module are mainly from the production of steel and aluminum. Carbon emissions of the wind farm module mainly come from the acquisition of steel including steel plate and steel rebar to produce the tower and base. Carbon emissions of the energy storage modules are from the production and manufacture of energy storage batteries and the manufacture of a prefabricated cabin. As for the building module, major carbon emission sources are the acquisition of cement and steel building materials as the raw material of the building.

#### 4.2.2. Calculation and Analysis in the Construction Phase

Carbon footprints in the construction phase include carbon emissions generated during the transportation and construction processes. Transportation is generally conducted by heavy duty trucks (maximum load as 46 t), and carbon emissions during the transportation process can be estimated with transportation distance and vehicle carbon emission factors, as in Table 3.

During the installation process, carbon emissions come from the energy consumed by using equipment for onsite installation. Electricity and water consumption are estimated based on construction equipment electricity demand, building construction water demand, and the project fire control requirements. In general, during the construction phase, the average power requirement is set as 300 kW with 10 h per day, and the water requirement is 3000 t per day.

The loss of carbon sinks due to the destruction of vegetation during construction is considered in the vegetation module. The construction period of the wind farm IES is 6 months, and the construction process occupied a total land area of 204,500 m^2^, in which the permanent land area is 15,500 m^2^ and the temporary land area is 189,000 m^2^, as shown in Table 6. Temporary land is mainly used for the construction of temporary facilities and wind turbine installation sites, while the permanent land is used for the installation of substation, wind turbine foundations, box transformer foundations, etc. The vegetation damaged area is the sum of the permanent land and the temporary land for the project during the construction phase. The vegetation destruction considered in this phase makes the carbon sequestration effect of the original vegetation disappear, and the resulting carbon sink loss should be calculated in carbon emissions. During the construction phase, 50,000 m^2^ of farmland was destroyed, 199,500 m^2^ of grassland was destroyed, resulting in equivalent 4.79 t carbon emissions. Table 7 summarizes the details of carbon sink losses during the construction phase.

Human activities cause carbon emissions in terms of the use of equipment, on-site living and on-site commuting by the construction human labor. The estimated average number of on-site construction workers is 50 people per day. With a construction period of 6 months and human ecological footprints 7.38 t CO_2_e/(year·per person) in China [40], carbon emissions of construction workers are calculated.

According to the actual construction project information, the carbon emissions in the construction phase are calculated as shown in Table 8. The energy consumption is the primary carbon emission source during the construction phase, while human activity is the second largest source. In total, these two account for 94.68% of the carbon emissions.

#### 4.2.3. Calculation and Analysis in the Operation and Maintenance Phase

The carbon emissions in the operation and maintenance phase mainly come from the electricity consumption, maintenance consumables, and fuel consumption generated in each module. During the operation process, since the electricity within the IES comes from wind power, there is no carbon emission as green power under normal operation circumstances. The maintenance typically occurs in the overhaul and replacement of small parts, taking 15% for the design life, and the carbon emissions are supposed to be 15% of what is produced in the production and materialization phase [41]. For a special large component wind blade, we consider the replacement of one blade per wind turbine within the life span [41].

For the transformer module, emissions originating from backup electricity consumption and SF_6_ leakage during the operation process and product replacement and vehicle fuel consumption during the maintenance process are considered: (1) the operation electricity consumption is caused by substation electrical equipment and station load. For a special situation when wind generation and energy storage cannot meet the power demand, a local power grid is required as a backup supply. The annual power consumption of the IES is about 300 MWh, and backup electricity should support averagely 5% of the total demand, leading to equivalent carbon emission of 300 t with a power grid carbon emission factor of 0.5810 t CO_2_/MWh. (2) Leakage of SF_6_ during the substation operation phase is considered as 0.5% of SF_6_ consumed in the full set of GISs in the typical design scheme of a 110 kV substation [29]. With the SF_6_ consumption in GISs as 2.25 t and the global warming potential of SF_6_ 23,900 times than that of CO_2_, the equivalent carbon emission of SF_6_ leakage is 5377.5 t in its life time. (3) Product replacement during maintenance is supposed to be 15% of all transformer-related components (the transmission lines and towers are assumed with no replacement), and thus carbon emissions are 15% of the those from the production and materialization phase. (4) Assuming that the maintenance frequency of traditional substations is four times a month, the average distance between the substation and the operation and maintenance center is 40 km, and one light duty truck is required for one maintenance task. There are thus 48 round trips per year, which result in 25.65 t carbon emissions in the project life span.

For the wind farm module, the product replacement and vehicle fuel consumption during the maintenance process are considered: (1) The carbon emissions of replacing one blade per turbine in a life span are calculated as in the production and materialization phase; (2) The carbon emissions of replacing other parts are 15% of those in the production and materialization phase; (3) Maintenance for wind farm is supposed to be twice a year with one light duty truck and 80 km per round trip.

For the storage module, since the containerized energy storage system requires minor maintenance in the life span, the carbon emissions are treated as 0. For the building module, the electricity consumption and maintenance vehicle fuel consumption are combined with the transformer module, and only 15% replacement of building small parts including glass and strand board are considered. For the vegetation module, destruction includes the land occupied during the operation and maintenance phase, and recovery includes the greens planted in the station. The permanent occupied land area 15,500 m^2^ grass land results in a carbon sink loss of 14,570 kg CO_2_e (15,500 × 0.047 × 20), and temporary 189,000 m^2^ land recovered in total with the1000 m^2^ greens planted in station results in 177,700 kg CO_2_e (5000 × 0.038 × 20 + 185,000 × 0.047 × 20) carbon emission offset. For the human activity module, 10 employees are considered in the IES project, including two management personnel and eight maintenance personnel, working 8 h on weekdays. The human ecological footprint data 7.28 t/(year·per person) in [41] is taken proportionally, thus the total carbon emission of 346.66 t (7.28 × (5/7) × (1/3) × 10 × 20) for human activity module in this phase is obtained.

The carbon emissions of all modules in the operation and maintenance phase are summarized in Table 9. The carbon emissions mainly come from SF_6_ leakage (67.80%), maintenance produced by the replacement of the wind farm (24.49%), and human activities (4.37%). The proportions of other projects in this phase are relatively small.

#### 4.2.4. Calculation and Analysis in the Disposal and Recycling Phase

According to the system boundary defined in Section 3.1, the disposal and recycling phase includes the process of equipment dismantling, material recycling, and vegetation recovery. When the life span expires, modules such as the transformer, wind farm and building will be dismantled, and a large amount of material will be discarded, landfilled or recycled. After the substation and wind farm are abandoned, the permanent land used for the project will be changed back to the original vegetation type.

The carbon emissions of the transformer module in this phase include electricity consumption in the recycling process of copper and steel. The transmission lines and towers are kept for future potential use and will not considered in the recycling process. Since the recovered materials can be reused, their carbon emissions are negative in the calculation. Carbon emissions of the wind farm module mainly come from energy consumption in the dismantling process and the recovery of steel, copper and aluminum. Carbon emissions of the building modules consist of the energy consumption during the dismantling and the offset from recycling the steel. Carbon emissions of lithium iron phosphate batteries are small, and the recycled steel offsets the carbon emissions generated in other stages.

Since the wind power IES has just been put into operation and is far from being recycled, there are a lack of relevant data on the recycling and disposal stage. This paper refers to the disposal situation in other cases for analysis. Many scholars claim that the carbon emissions at the demolition stage can be approximately equal to 10% of the construction stage [42]. According to the China Association of Circular Economic, the average recycling rate of domestic steel is about 85%, the average recycling rate of aluminum is about 76%, and the recycling rate of copper is about 90%. In addition, considering that various metal equipment will suffer from corrosion and loss in the long life-span, this paper sets loss factor as 50% for the recycling of various metal materials. Therefore, we have 42.5% of steel, 38% of aluminum, and 45% of copper in each module are recycled as raw materials for metal smelting and processing. Landfill disposal is carried out for other materials such as concrete, waste resin, and scrap blades. This paper considers that the permanent occupied land 15,500 m^2^ would return to grassland within one year in the disposal and recycling phase, thus 0.73 t of carbon emissions are offset. For human activities, it is assumed the carbon emissions are 10% of those in the construction phase, i.e., 18.45 t.

Table 10 summarizes the carbon emissions of related materials and energy in the disposal and recycling phase. Carbon reduction mainly comes from the steel recycling of the wind power module. After considering the recycling and utilization of metal materials, the total carbon emissions of the substation module, wind farm module and energy storage module are all negative, i.e., the offset carbon emissions are greater than the generated carbon emissions.

### 4.3. Total Lifecycle Carbon Footprint of the Wind Power IES

#### 4.3.1. Calculation and Analysis of the Lifecycle Carbon Footprint of the IES

Based on the above calculation and related data query, the total carbon footprints of each module at each phase in the IES are shown in Table 11.

**Figure 4:**
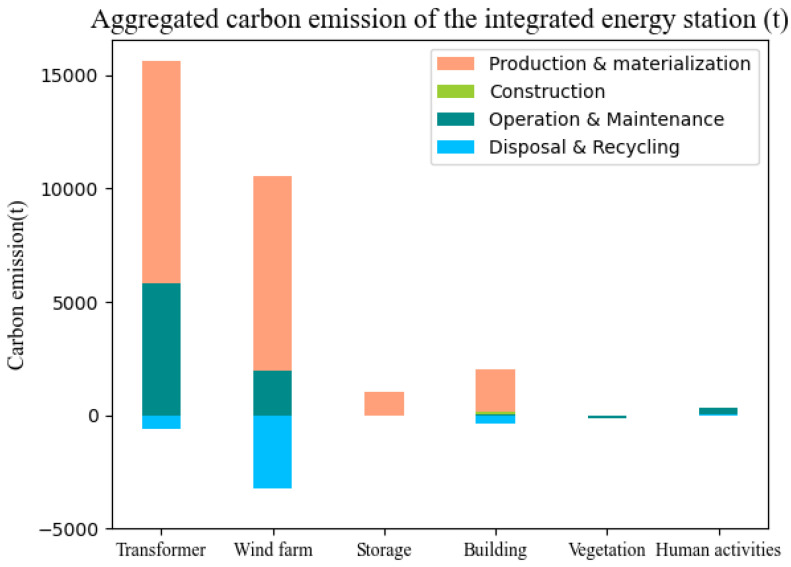
Aggregated carbon emission of the IES.

Figure 4 shows carbon emissions of each module in the lifecycle for the studied IES. Carbon emissions generated by the transformer and wind farm modules account for the largest proportion of the entire IES carbon emissions. The building module is the third carbon emission source in the IES, followed by the human activity module. Energy storage and vegetation contribute the least to the carbon emissions. The lifecycle carbon emissions of the main transformer module are 20,971.57 t, accounting for 62.52% of the total carbon emissions of the IES. The lifecycle carbon emissions of the wind farm, building, and storage module account for 28.05%, 5.27%, and 2.99%, respectively.

**Figure 5:**
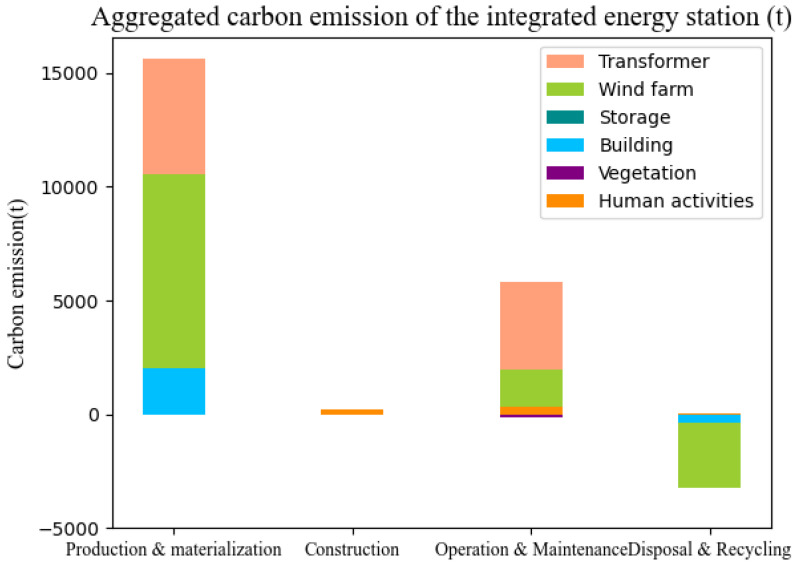
Carbon emissions of each phase in the IES.

Figure 5 shows the carbon emissions generated in each phase of the IES in the lifecycle. Production and materialization is the main phase of carbon emissions, releasing 87.21% of the total carbon emissions; the second largest source of carbon emissions of the IES is the operation and maintenance phase, accounting for 23.65% of the total carbon emissions. The carbon emissions in the construction phase are 1.87% of the total carbon emissions. The carbon emissions in the disposal and recycling phase of the IES includes two parts. On one hand, the carbon emissions generated during the demolition work is 62.32 t; on the other hand, the recycling and utilization of various metal resources such as copper, steel, and aluminum make up a large amount of carbon emissions, a corresponding reduction of 4331.87 t of carbon emissions. Combining the two data sources, it is concluded that the net carbon emissions in the disposal and recovery stage is −4269.55 t, i.e., this phase can make up for 4269.55 t of carbon emissions generated in other phases.

The results of the wind power IES are compared to the results obtained in the reviewed literature. The levels of the total carbon emissions for modules and the IESs are different since the raw materials involved, construction management, and operation rules may differ among projects. For an individual module including transformer, wind farm, and building, the main influence factors derived from our study are quite similar to that reported in the literature. This is because the raw materials and design scheme are similar following the recent 110 kV substation and wind farm standard. Taking the IES as a holistic system, the most relevant sources of CO_2_ emission are the transformer and wind farms, which are consistent with the existing papers on wind power stations.

#### 4.3.2. Energy Saving and Emission Reduction Strategies

The application of the LCA method to analyze the carbon footprint of the IESs can objectively and comprehensively measure the project energy consumption and carbon emissions. It can provide a comprehensive data reference for evaluating the energy saving and emission reduction benefits for constructing new IESs or renovating existing substations. By analyzing IESs’ lifecycle carbon emissions, key influencing factors in each phase and each module can be identified, which can provide a valuable guideline or suggestions for carbon management studies.

The production and materialization phase typically provide the largest carbon emissions in the lifecycle of the IES. The key to the emission reduction and optimization in this phase lies in the optimization and upgrading of the production process in the upstream industries. At this stage, China is in a period of rapid economic development. Reducing carbon emissions during production and materialization will help China achieve the goal of carbon neutrality and is of great significance to China’s commitment to the Paris Agreement.

The carbon emissions in the operation and maintenance phase are second among all four phases in the LCA analysis. The carbon emissions caused by the SF_6_ leakage in the transformer module are the largest factor, accounting for 67.80% of the total carbon emissions in this phase. For this study case, SF_6_ leakage is calculated based on the worst case, taking the 0.5% leakage rate. With the development of GIS manufacturing quality, installation and maintenance technology, this part could be significantly reduced. Moreover, clean air GIS gas, composed of 80% nitrogen and 20% oxygen with no carbon emissions, can be considered as an alternative. If the clean air GIS is used in the IES project, the equivalent carbon emission caused by the leakage of SF_6_ gas can be significantly reduced.

Although the carbon emissions in the construction phase are much smaller than those in the production and materialization and the operation and maintenance phases, the carbon emissions in the construction phase are relatively concentrated. Energy consumption brings in a large proportion at 65.30%, while human activities account for 29.38%. In view of the carbon emissions caused by high-energy consumption construction, prefabricated construction for the entire station building can be considered. The use of prefabricated cabins instead of ordinary brick-concrete structures can significantly shorten the construction period, reduce the amount of engineering quantity, and save construction costs. Moreover, green construction and onsite education and management is preferred to instruct construction and on-duty workers to take ownership of their personal contribution to climate change.

The net carbon emissions in the disposal and recycling phase are negative, i.e., carbon emissions made up by the metal resources recycled in this phase not only offset the carbon emissions generated in the dismantling process, but also make up for the carbon emissions generated in other phases. The emission reduction strategy in this phase is to improve the utilization rate of resource recovery. Through the recycling of metal resources, it can not only solve the problem of resource shortages that may be faced in the future, but also effectively achieve energy conservation and emission reduction, and reduce pollution to the environment.

## 5. Conclusions

This paper develops a novel carbon footprint accounting model for the IESs based on LCA theory, and the model is applied to an IES project in China to calculate the carbon emissions. Based on the case study analysis results, carbon emission reduction strategies are provided. The main conclusions are summarized as follows:

(1) Among the four phases in LCA of the studied IES project in China, the carbon emission percentages from high to low are: production and materialization phase (87.21%), operation and maintenance phase (23.65%), construction phase (1.87%), and disposal and recycling phase (−12.73%). The production and materialization phase and operation and maintenance phase make up the majority of the project’s lifecycle carbon emissions; technology advancement in these two phases will bring significant potential to carbon reduction.
(2) In the production and materialization phase, the modules with carbon emissions from high to low are: transformer (53.49%), wind farm (36.10%), buildings (6.85%) and storage (3.56%). The transformer and wind farm are the key modules for IES’s carbon emission reduction in this phase. Sustainable materials and technologies used in production and manufacturing processes will make a great contribution to green IES development.
(3) In the operation and maintenance phase, the SF_6_ leakage in the transformer (67.8%) and wind farm part replacement (24.49%) are the key sectors of carbon emissions. Using transformers with less SF_6_ leakage, improving the product quality and extending the lifetime of wind turbines will achieve significant results for carbon reduction in this phase.
(4) The carbon emission offset effect in the disposal and recycling phase accounts for a significant proportion in the lifecycle carbon emission of IESs. Reuse and recycle wind farm (76.64%), transformer (14.52%) and buildings (9.39%) are the most important modules for reducing carbon emissions in this phase. Studies on recycling and reuse methods should be given attention by the government and IES owners.

The current research on carbon emissions for energy infrastructure projects in China is still in the early stage and faces the following challenges. Currently, there are no systematic carbon emission calculation standard and carbon emission factor database for the IES projects in China. Moreover, since multiple modules are interconnected in IESs, there are complexities and uncertainties in the calculation of carbon emissions, which have not been considered yet. In future studies, the uncertainties in renewable energy generation will be considered in the IES footprint accounting model, and more case studies will be investigated to gradually improve and standardize the lifecycle carbon footprint analysis for the IESs.

## Figures and Tables

**Table 2:** Parameters in the building module.

Building Name	Area (m^2^)	High (m)	No. of Floors	Notes
Comprehensive office building	1134	4.2	2	It consists of offices, conference rooms, reference rooms, lounges, dining hall, etc.
GIS room	216	5.5	1	It consists of main control room and secondary equipment room.
Auxiliary room	143	3.9	1	It consists of security office, fire pump room, domestic pump room and spare parts warehouse.

**Table 3:** Carbon footprint sources in the LCA of the wind power IES.

	Transformer	Wind Farm	Storage	Building	Human Activities	Vegetation
Production & Materialization	Manufacturing of transformers and related primary and secondary equipment	Manufacturing of wind turbine parts	Manufacturing of storage batteries and casings	Manufacturing of cement, steel, standard timber and other materials	-	-
Construction	Energy consumption of transportation and installation of transformer equipment	Energy consumption of transportation and installation of wind turbines	Energy consumption of transportation and installation of batteries and accessories	Energy consumption of transportation and construction	Energy consumption of construction workers on-site living and commuting	Carbon sink due to vegetation destruction
Operation & Maintenance	Backup power supply, equipment maintenance and replacement, and SF_6_ leakage	Inspection, repair and replacement of blade and parts	Inspection, equipment maintenance, and part replacement	Energy consumption during operation and part replacement	Energy consumption of on duty staff living, working and commuting in the station	Carbon sink due to vegetation destruction and greens planted in the station
Disposal & Recycling	Energy consumption of demolition and recycled metal	Energy consumption of demolition and recycled metal	Energy consumption of demolition and recycled metal	Energy consumption of demolition and recycled metal	Human carbon emissions during disposal	Vegetation restoration after project demolition

**Table 4:** Main materials and energy carbon emission factor/carbon sequestration rates.

Material	Carbon Emission Factor	Data Source
Steel	2050 kg CO_2_e/t	[35]
Steel rebar	2340 kg CO_2_e/t	[35]
Steel plate	2400 kg CO_2_e/t	[35]
Copper	6836 kg CO_2_e/t	[35]
Aluminum	20,300 kg CO_2_e/t	[35]
Cement	735 kg CO_2_e/t	[35]
Concrete	385 kg CO_2_e/m^3^	[35]
Sand	2.51 kg CO_2_e/t	[35]
Stone	2.18 kg CO_2_e/t	[35]
Brick (240 mm × 115 mm × 53 mm)	134 kg CO_2_e/m^3^	[35]
Glass	1130 kg CO_2_e/t	[35]
Glass fiber	2100 kg CO_2_e/t	[35]
Polystyrene	4620 kg CO_2_e/t	[35]
Polyurethane	5220 kg CO_2_e/t	[35]
Tap water	0.168 kg CO_2_e/t	[35]
Lubricating oil	71.87 t CO_2_e/TJ	[35]
Light duty gas trucks (maximum load 2 t)	0.334 kg CO_2_e/(t·km)	[35]
Heavy duty diesel trucks (maximum load 46 t)	0.057 kg CO_2_e/(t·km)	[35]
SF_6_	23,900 kg/kg	[29]
Power grid	0.5810 t CO_2_/MWh	[36]
Grassland carbon sequestration capacity	0.047 kg/m^2^·per year	[37]
Farmland carbon sequestration rate	0.038 kg/m^2^·per year	[38]

**Table 5:** Carbon emissions in the production and materialization phase.

Module	Project	Material	Content	Carbon Emissions (t)	(%)
Transformer	Main transformer	Copper	21.69 t	148.27	0.51
		Steel	86.76 t	177.86	0.61
	14 box-type and station transformers	Copper	72.8 t	497.66	1.70
		Steel	291.2 t	596.96	2.04
	Transmission lines	Copper	85 t	581.06	1.99
		Aluminum	280 t	6347	21.70
		Steel	3560 t	7298	24.95
Wind farm	Blade	Steel	460 t	943	3.22
		Glass fiber	482 t	1012.2	3.46
	Tower	Steel plate	1920 t	4608	15.75
	Base	Steel rebar	490 t	1146.60	3.92
		Concrete	4200 m^3^	1617	5.53
	Hub	Steel	277.20 t	568.26	1.94
		Copper	50.40 t	344.53	1.18
		Aluminum	3.780 t	76.73	0.26
		Glass fiber	28 t	58.8	0.20
		Plastic-Polystyrene	20.43 t	94.39	0.32
		Coating-Polyurethane	17.43 t	90.98	0.31
		Lubricating oil	4.83 t	0.35	0.00
Storage	Energy storage battery		4.4 MWh	950.4	3.25
	Steel plate (prefabricated cabin)		37.45 t	89.88	0.31
Building	Cement		1292 t	949.62	3.25
	Steel rebar		403 t	943.02	3.22
	Sand		2185 t	5.48	0.02
	Stone		4355 t	9.49	0.03
	Brick		12,135 blocks	2.22	0.01
	Glass		25 t	28.25	0.10
	Strand board		323 m^3^	65.12	0.22

**Table 6:** Permanent and temporary site area (m^2^).

Project	Permanent Occupied Area	Temporary Occupied Area	Total
Wind turbine and installation site	5100	22,900	28,000
110 kV substation	10,200	-	10,200
Collector circuit	200	11,200	11,200
Wind farm maintenance road	-	146,900	146,900
Construction production and living site	-	8000	8000
Total	15,500	189,000	204,500

**Table 7:** Carbon sinks caused by vegetation destruction during the construction phase.

Process	Original Land Type	Change Area (m^2^)	Change Time (Year)	Unit Carbon Sequestration (kg/m^2^·per Year)	Carbon Emission (t CO_2_e)
Vegetation destruction	Farmland	−5000	0.5	0.038	−0.10
Vegetation destruction	Grassland	−199,500	0.5	0.047	−4.69
Total	-	−204,500	0.5	-	−4.79

**Table 8:** Carbon emissions in the construction phase.

Category	Module	Content		Carbon Emission (t CO_2_e)	(%)
Transportation		Loading weights, No. of round trips	One-way distance (km)		
	Transformer	4397.45 t, 100	250	2.85	0.45
	Wind farm	14,254.07 t, 330	650	24.45	3.89
	Storage	37.45 t, 6	250	0.17	0.03
	Building	8714.36 t, 200	50	1.14	0.18
Construction and installation	Vegetation	Carbon sink loss	4.79 t	4.79	0.76
	Human activities	Ecological footprints	7.38 t (year·per person)	184.5	29.38
	Total energy consumption	Electricity	547.5 MWh	318.10	50.65
		Water	547,500 t	91.98	14.65

**Table 9:** Carbon emissions in the operation and maintenance phase.

Module	Sector	Content	Carbon Emission (t CO_2_e)	(%)
Transformer	Backup electricity	(5% × 300 × 20) MWh	174.30	2.20
	SF6 leakage	2.25 × 0.5% t/year	5377.50	67.80
	Product replacement	15% of the parts	213.11	2.69
	Maintenance vehicle	40 km one way, 4/month	25.65	0.32
Wind farm	Wind blade replacement	One blade per turbine in life span	651.73	8.22
	Replacement other parts	15% of the parts	1290.85	16.27
	Maintenance vehicle	40 km one way, 2/year	1.07	0.01
Storage	Maintenance	Not considered	0	0.00
Building	Maintenance replacement	15% of small parts	14.00	0.18
Vegetation	Destruction	15,500 m^2^ grass land	14.57	0.18
	Recovery	Temporary 189,000 m^2^ land recovered, 1000 m^2^ greens planted	−177.7	−2.24
Human activities	On-duty human activity	1.73 t/(year·per person)	346.66	4.37

**Table 10:** Carbon emissions in the disposal and recycling phase.

Category	Module	Project	Content	Carbon Emission (t)	(%)
Dismantling		Electricity	54.75 MWh	31.81	−0.75
		Water	54,750 t	9.20	−0.22
		Transportation	-	2.86	−0.07
	Human activities	Ecological footprints	10% of those in construction phase	18.45	−0.43
Recycling	Transformer	Copper	42.52 t	−290.67	6.81
		Steel	160.63 t	−329.30	7.71
	Wind farm	Cooper	22.680 t	−155.04	3.63
		Aluminum	1.44 t	−29.16	0.68
		Steel	313.31 t	−642.29	15.04
		Steel plate	816 t	−1958.40	45.87
		Steel rebar	208.25 t	−487.30	11.41
	Storage	Steel plate	15.92 t	−38.20	0.89
	Building	Steel rebar	171.7 t	−400.78	9.39
	Vegetation	Land recovery	15,500 m^2^	−0.73	0.02

**Table 11:** Carbon emissions (t) in each phase and module in LCA.

Module	Production & Materialization	Construction	Operation & Maintenance	Disposal & Recycling	Total	(%)
Transformer	15,646.81	139.54	5790.56	−605.35	20,971.57	62.52
Wind farm	10,560.84	161.15	1943.65	−3257.57	9408.07	28.05
Storage	1040.28	0.17	0	−38.2	1002.25	2.99
Building	2003.2	137.83	14	−386.16	1768.88	5.27
Vegetation	0	4.79	−163.13	−0.73	−159.07	−0.47
Human activities	0	184.50	346.66	18.45	549.61	1.64
Total	29,251.13	627.984	7931.74	−4269.55	33,541.30	100.00
(%)	87.21	1.87	23.65	−12.73	100	

## Data Availability

Not applicable.
